# Autologous blood transfusion promotes autophagy and inhibits hepatocellular carcinoma progression through HIF‐1α signalling pathway

**DOI:** 10.1111/jcmm.17736

**Published:** 2023-04-10

**Authors:** Yu Bai, Tong Liu, Ying‐Hui Cui, Zhen‐Zhou Li, Xiao‐Fang Zhou, Yong Cheng, Jin‐Huo Wang, Jian‐Rong Guo

**Affiliations:** ^1^ Graduate School of Wannan Medical College Wuhu Anhui China; ^2^ Department of Anesthesiology, Shanghai Gongli Hospital Naval Military Medical University Shanghai China

**Keywords:** autologous blood transfusion, autophagy, hepatocellular carcinoma, HIF‐1α signalling pathway

## Abstract

To explore the molecular mechanism of autologous blood transfusion promoting autophagy of hepatocellular carcinoma (HCC) cells and inhibiting the HCC progression through HIF‐1α signalling pathway. This is a research paper. Rat hepatocellular carcinoma model and HepG2 cell model were built. The rats with HCC were conducted a surgery, and their blood was collected for detection to detect the recurrence and metastasis of the rats. Western blot was used to analysed the expression of HIF‐1α, TP53, MDM2, ATG5 and ATG14 protein. The apoptosis rate of HepG2 cells was detected by flow cytometry, and autophagosomes were observed by transmission electron microscopy. HIF‐1α expression was measured by immunofluorescence assay. The expressions of HIF‐1α, TP53, MDM2, ATG5 and ATG14 protein were highest in model + autoblood group compared with the model group. HIF‐1α content of model group was higher, but content of TP53, MDM2, ATG5 and ATG14 in the model group is the second. The highest apoptosis rate was found in HepG2 + autoblood group. The number of autophagosomes in HepG2 + autoblood was obviously larger than that of HepG2 + autoblood + inhibitor. HIF‐1α expression of immunofluorescence assay showed that high expression of HIF‐1α was clearly observed in HepG2 and HepG2 + autoblood group from confocal observation. However, there was no HIF‐1α protein expression in HepG2 + autoblood + inhibitor group. The migration rate in HepG2 group, HepG2 + autoblood group and HepG2 + autoblood + inhibitor group was 85.71 ± 7.38%, 14.36 ± 6.54% and 61.25 ± 5.39%, respectively. Autologous blood transfusion promotes autophagy of HCC cells through HIF‐1α signalling pathway, which further inhibits HCC migration and erosion.

## 1 INTRODUCTION

Hepatocellular carcinoma (HCC) is a widely malignant tumour in the world, which seriously threatens human life and health. HCC has insidious onset, low early diagnosis rate, rapid progression, high degree of malignancy, poor prognosis and easy recurrence and metastasis.^1^ Although the basic and clinical studies of this disease have made great progress in recent years, the effect of its prevention and treatment is still not satisfactory. HCC is a common tumour of the digestive system, and whether radical resection can be performed largely determines its prognosis. It has been reported that the liver cancer patients with the 5‐year survival rate undergoing radical resection can reach more than 70%.^2^ However, the liver is rich in blood supply and complicated in anatomy, and tumour growth sites are different and often invade the large intrahepatic vessels. Therefore, the implementation of standard liver resection often encounters the problem of massive intraoperative blood loss. At present, the common methods of blood transfusion include allogeneic blood transfusion and autologous blood transfusion. Allogeneic blood transfusion often has the problem of blood source tension and is prone to immunosuppression. Storage autologous blood transfusion, through preoperative blood storage and intraoperative blood transfusion, can theoretically reduce the concentration of red blood cells in the intraoperative blood loss, control the actual blood loss, avoid numerous of allogeneic blood transfusion and reduce the corresponding complications.^3^

Autophagy is the formation of autophagosomes by cytoplasm or organelles in the phagocytic body after cell stimulation. The contents of autophagosomes were degraded and reused by lysosome.^4^ Autophagy occurs under many physiological and pathological conditions in living organisms. It refers to the dynamic changes in the membrane structure of cells, resulting in the degradation of intracellular proteins and organelles.^5^ In terms of evolution, autophagy is a highly conserved phenomenon. Through its degradation and circulation, it produces nucleic acids, amino acids, fatty acids, sugars and ATP, which are used to support cell metabolism and survival in starvation state.^6^, ^7^ At the same time, autophagy can also remove protein aggregates and damaged organelles, and maintain the quality of proteins and organelles.^8^

Autophagy also plays an essential role in the occurrence and development of tumours. Studies have indicated that autophagic cell death plays a dual role in the survival and death of both normal and cancer cells. On the one hand, autophagy can initiate the normal cell death process to accelerate tumour death and inhibit tumour growth. On the other hand, autophagy can act as an adaptive mechanism of cancer cells to protect tumour cells from various nutritional deficiencies or the erosion caused by chemotherapy drugs.^9^ Therefore, it has important significance to research the role of autophagy in tumorigenesis and the role of autophagy in the action of chemotherapy drugs for clinical treatment of cancer.

The tumour is always in a microenvironment of relative ischemia and hypoxia.^10^ Under the hypoxia, cells form autophagy‐lysosome which can degrade macromolecular substance in the cytoplasm (such as proteins and RNA, and excessive glycogen storage) and some cell's endogenous substrates (including damaged organelles) to realize recycling. This process can maintain the stability of cells themselves, so as to realize the metabolism of the cell and some organelles.^11^, ^12^ Hypoxia‐inducible factor 1 (HIF‐1α) is an essential transcription factor activated in hypoxia environment, and its oxygen‐regulated subunit HIF‐1α regulates the expression of chemotherapeutic resistance gene MDR1, which has received increasing attention. Under hypoxia, tumour cells can improve their ability to adapt to hypoxia through a series of biological behaviours, among which HIF‐1α and autophagy are more important.^13^, ^14^ Previous studies on chondrocytes have shown that with the increase of expression level of HIF‐1α in chondrocytes, the level of autophagy is also significantly increased, suggesting that there is a positive correlation between the expression level of HIF‐1α in chondrocytes and autophagy.^15^

In recent years, many studies have shown that autophagy is closely related to the occurrence, development and recurrence of hepatocellular carcinoma. In particular, the molecular mechanism of whether autologous blood transfusion can affect autophagy to inhibit the malignant progression of hepatocellular carcinoma has not been reported. Therefore, the mechanisms related to autophagy and liver cancer need a further research to provide a new basis for finding more effective targets for hepatocellular carcinoma treatment.

## 2 EXPERIMENTAL SECTION

### 2.1 Materials

Sixty healthy SD rats (200–250 g), half male and half female, were purchased from experimental animal centre of Harbin Medical College. HIF‐1α inhibitor (Oltipraz; CS‐7392) was purchased from Innochem (Beijing, China). PBS, FBS and DMEM were purchased from Gibco (Shanghai, China). HIF‐1α, TP53, MDM2, ATG5, ATG14 protein, rabbit anti‐rat primary antibody β‐actin and HRP‐labelled sheep anti‐rabbit secondary antibody were purchased from Beyotime Biotechnology. HepG2 cell (ATCC HB‐8065) was purchased from ThermoFisher Scientific company.

Domestic self‐2000 blood recovery machine (Wandong Kangyuan); Transmission electron microscopy (TEM; JEOL‐1000, Japan); Centrifuge (5430 R, Eppendorf); Rotary evaporators (EYELA); Automatic microplate reader (SYNERGY HTX, BioTek); Flow cytometry (SN AW29005, CytoFLEXS, Beckman Coulter); Delta Vision Ultra (C3655‐157, General Electric Company).

### 2.2 Methods

#### 2.2.1 Construction of rat hepatocellular carcinoma model

After 1 week of normal feeding to adapt to the environment, SD rats were randomly divided into four groups. One group was healthy rats. HepG2 cells were injected subcutaneously into the other three groups. The tumorigenesis of rats was observed and recorded every 3 days. The rat model of liver cancer was established after 42 days. The experimental groups were numbered as normal group, liver cancer model group 1, liver cancer model group 2 and liver cancer model group 3, respectively.

#### 2.2.2 Operation of the hepatocellular carcinoma rats

Abdominal surgery was performed to remove visible hepatocellular carcinoma tissues of the three groups of hepatocellular carcinoma model rats, respectively, and the resected tissues were preserved fresh for subsequent experiments to separate hepatocellular carcinoma cells. At the same time, the blood was recovered from the surgical field of each rat by the domestic autologous −2000 blood recovery machine. After excision of hepatocellular carcinoma tissue, the wound was sutured with suture. Then, 10 mL normal saline was injected into the normal mice by tail vein and each rat in hepatocellular carcinoma model group 1. Each rat in hepatocellular carcinoma model group 2 was injected with 10 mL recovered autologous red blood cells from tail vein. Each rat in hepatocellular carcinoma model group 3 was intravenously injected with autologous red blood cells (10 mL) + HIF‐1A inhibitor (Oltipraz). All rats were fed routinely for 8 weeks.

#### 2.2.3 HIF‐1α, TP53, MDM2, ATG5 and ATG14 expression

Western blot was used to analyse the expression of HIF‐1α, TP53, MDM2, ATG5 and ATG14. After discarding the cell culture medium, the cells were washed with pre‐cooled PBS for three times. Protease inhibitor was added to cell lysis solution RIPA in a ratio of 1:100 and placed on ice for 30 min. The protein is then scraped off with a cell scraper and put in the EP tube. The protein solution was centrifuged with 12,000 *g* at 4°C for 15 min with high speed; then, the precipitation was discarded to collect the supernatant. The protein samples were mixed with 5 × SDS loading buffer 4:1 and incubated at 95°C in metal bath for 10 min to denaturate the proteins. Sample 30 μg was added in each lane, and electrophoresis was performed at constant pressure of 80 V for 30 min. Proteins were transferred to NC membrane and sealed with TBST solution containing 5% BSA for 2 h. After sealing, primary antibodies [Rabbit anti‐rat primary antibody HIF‐1α, TP53, MDM2, ATG5, ATG14 (1:500), rabbit anti‐rat primary antibody β‐actin (1:1000)] were incubated overnight. The membrane was then washed with TBST for three times and incubated with secondary antibodies (HRP‐labelled sheep anti‐rabbit secondary antibody). The membrane was washed for three times after shaking for 1 h. According to the instructions, liquid A and liquid B in the luminescent solution were mixed 1:1 and uniformly added to the PVDF membrane. The imaging system was run under the protection of light, and the protein expression information was collected.

#### 2.2.4 Construction of HepG2 cell model

HepG2 cells were incubated in the DMEM with 10%FBS at 37°C. Generally, cells are subcultured when the cell density reaches about 90%. The HepG2 cells were washed with PBS twice, and digested with trypsin, then incubated at 37°C for 2 min. After the trypsin being sucked out and the cells being suspended in complete medium, the cells were blown into a single cell suspension for further experiment.

Red blood cells from autologous recovery were used in autologous‐blood recovery machine during the operation. The intraoperative blood was recovered to the blood storage tank through a negative pressure suction device and mixed with appropriate anticoagulant during the suction process. After multi‐layer filtration, the red blood cells are separated by a high‐speed centrifugal pump, and the waste liquid, broken cells and harmful components are shunted into the waste liquid bag. Finally, the red blood cells can be washed and concentrated with normal saline to obtain concentrated red blood cells with a specific volume of 50%–65%. Group 1: HepG2 + saline (100 μL/100 μL); Group 2: HepG2 + autoblood, red blood cells from autologous recovery/HepG2 (100 μL/100 μL); Group 3: HepG2 + autoblood + inhibitor, HIF‐1α inhibitor (Oltipraz; 10 μL). All cells in different groups were incubated at 37°C incubator.

#### 2.2.5 HepG2 cell apoptosis

The HepG2 cells in the three groups were incubated at 37°C with CO_2_ for conventional sterile culture for 14 days. The cells in three groups were collected and digested by trypsin without EDTA. The concentration of cell was adjusted according to the experimental needs, and the groups were marked on the aseptic flow tube. The cells in each group were treated according to the standard AV‐PI double staining procedure and detected by flow cytometry.

#### 2.2.6 Autophagosomes observation by transmission electron microscopy

HepG2 cells of each group were collected, and autophagosomes in cells were analysed by transmission electron microscopy. The cells were washed twice by pre‐cooled PBS and digested by trypsin; then, they were centrifuged at 800 rpm for 5 min to remove cell fragments. The cells were re‐suspended with PBS and centrifuged at 1500 rpm for 10 min. The cells were fixed with 2.5% glutaraldehyde for 2 h. After fixation, samples were washed with buffer solution for 20 min. The specimens were dehydrated by gradient dehydration with 70%, 80% and 90% acetone for 15 min and 100% acetone twice for 10 min each time. The sample was buried in a porous rubber embedded plate to prepare 45 nm ultrathin section and stained by lead acetate. Images of samples were taken using HITACHI 7650 transmission electron microscope.

#### 2.2.7 HIF‐1α expression of immunofluorescence assay

HepG2 cells were collected and washed with PBS for three times. The cells were drilled with 3% Triton for 20 min and closed with 5% BSA for 30 min. The primary antibody (diluted 1:100) was added. After removing the primary antibody, the membranes were incubated with the secondary antibody (diluted: 1:200) for 2 h at room temperature in a dark place. The membranes were washed with TBST for three times to remove sencondary antibody. DAPI was used to stain nuclear for 1 min and sealed the membranes. A fluorescence microscope was used to observe the images.

#### 2.2.8 Migration and invasion of HepG2 cells

Cell invasion ability was observed by Transwell assay. Matrigel glue was diluted with DMEM in the ratio of 1∶36, and 100 μL was added in the upper chamber; then, the cells were incubated at 37°C for 3 h. Cells were taken from each group and digested with trypsin to prepare the cell suspension with a cell density of 1 × 10^6^/mL. 200 μL of cells was added in a lower chamber and incubated in the DMEM with 15% FBS for 24 h. The chamber was taken out, fixed with formaldehyde for 20 min and stained with 0.1% crystal violet for 20 min. The chamber was rinsed with double steam water. The cells on the surface of the upper chamber were removed with cotton swabs and observed under an inverted microscope.

Cell migration ability was observed by cell scratch test. Cells were digested and cultured in 6‐well plates with the concentration of 1 × 10^6^/mL/well. After 2 h, the cells were completely overgrown, and the cells were starved in serum‐free DMEM medium for 12 h. 20 μL sample tip was used to scratch along the scale of the 6‐well plate. The suspended cells were washed with PBS and added with fresh medium containing 1% serum for routine culture. At 0 and 72 h, the migration of cells in each group was observed and the width of scratches was measured to calculate the cell migration rate. Cell migration rate = (0 h scratch width − 72 h scratch width)/0 h scratch width × 100%.

#### 2.2.9 Western blot analysis of HIF‐1α, TP53, MDM2, ATG5 and ATG14 expression in HepG2 cells

HepG2 cells from each group were collected, and Western blot was used to analyse the expression of HIF‐1α, TP53, MDM2, ATG5 and ATG14. Specific procedure was shown in 2.2.4.

### 2.3 Statistical analysis

After data collection and summary, statistical professionals of the Mathematical Statistics Teaching and Research Office of the University were entrusted to be responsible for data analysis and processing, and SPSS20.0 statistical software was used to perform the data statistical analysis. The results were analysed by one‐way analysis of variance (anova) and expressed as the mean ± standard deviation (SD). *p* < 0.05 was considered as statistical significance.

## 3 RESULTS

### 3.1 Western blot analysis of HIF‐1α, TP53, MDM2, ATG5 and ATG14 expression

**FIGURE 1:**
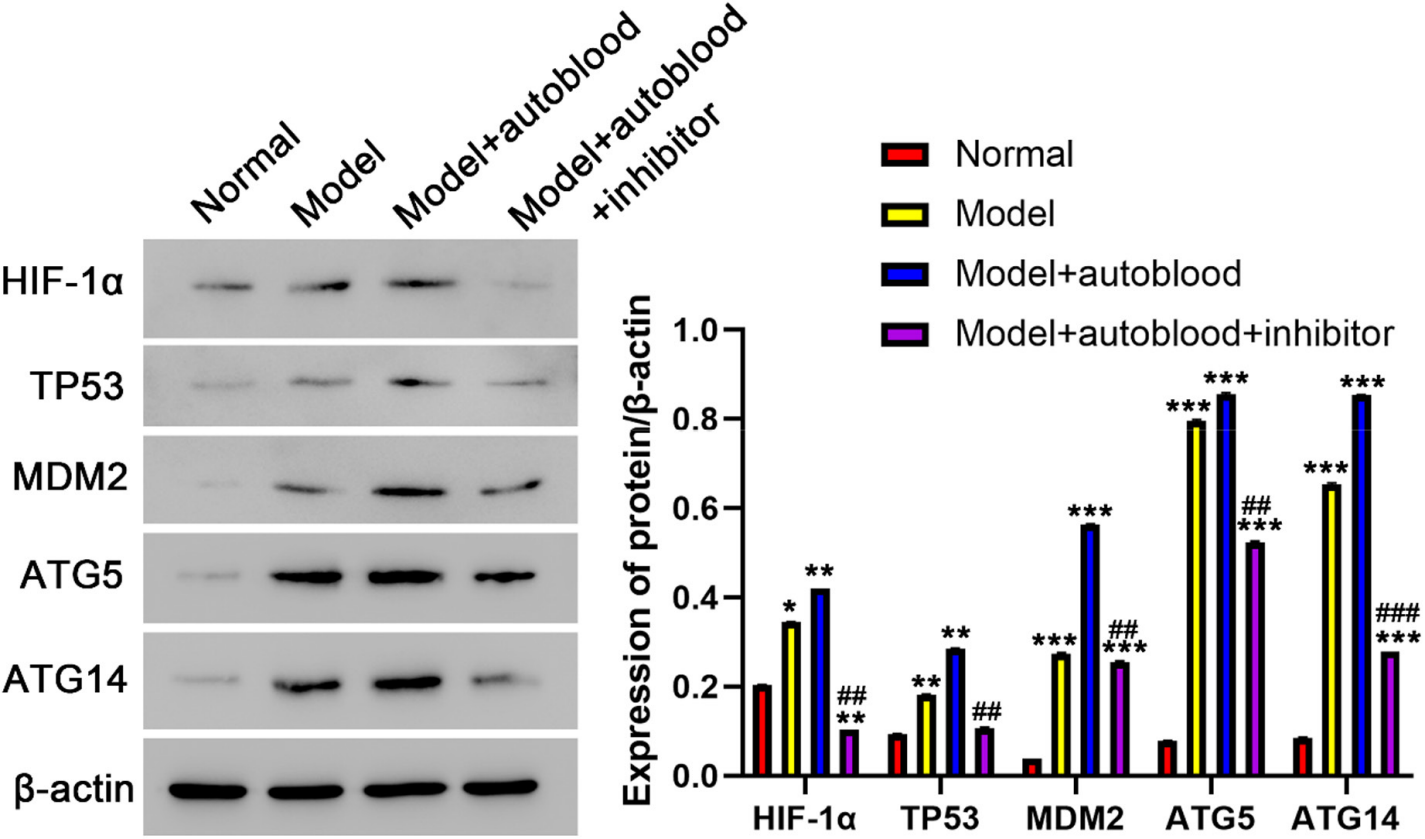
Protein levels of HIF‐1α, TP53, MDM2, ATG5, ATG14 in different groups determined by Western blot (Mean ± SD, *n* = 3). *p < 0.05; **p < 0.01; ***p < 0.001 compared with normal group; ^*###*^ *p* < 0.001; ^*##*^ *p* < 0.01 compared with model group.

The expressions of HIF‐1α, TP53, MDM2, ATG5 and ATG14 proteins in liver tissue of rats were showed in Figure 1. The expressions of HIF‐1α, TP53, MDM2, ATG5 and ATG14 protein were highest in model + autoblood group, and the second was the model group. There was a significant difference, compared with the normal group, *p < 0.05; **p < 0.01; ***p < 0.001. Compare with model group, ^*###*^ *p* < 0.001; ^#*#*^ *p* < 0.01.

### 3.2 HepG2 cell apoptosis

**FIGURE 2:**
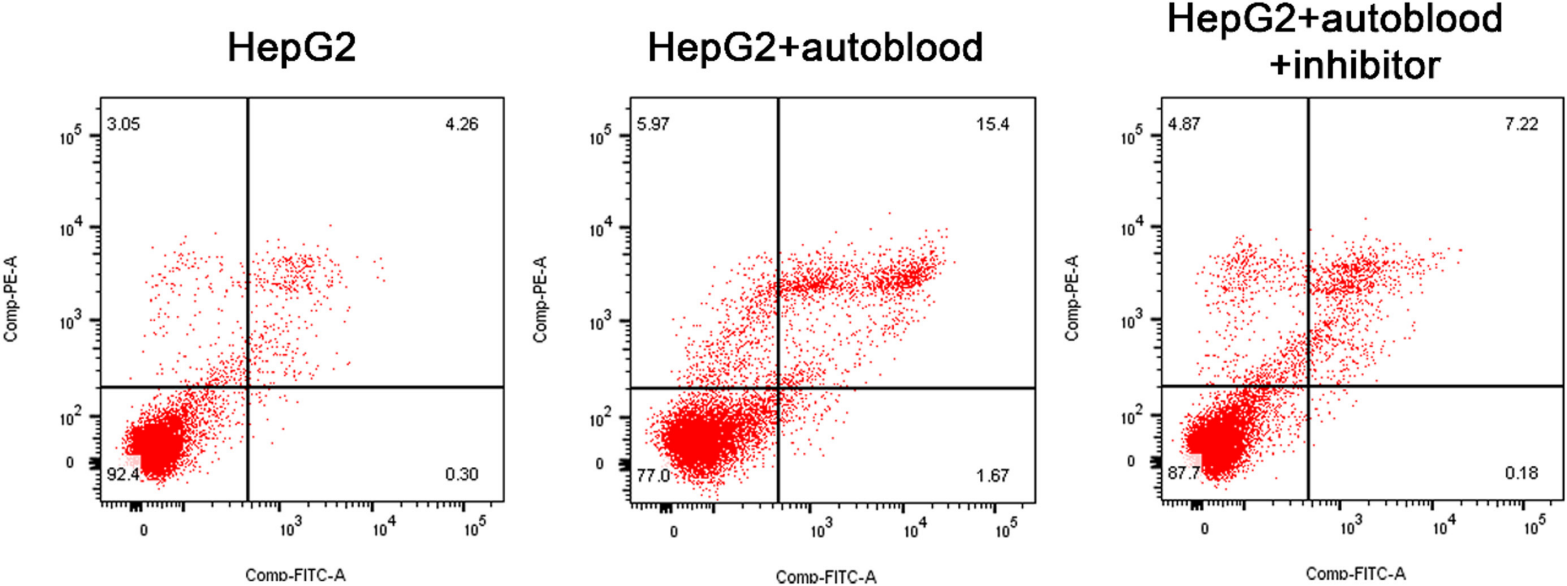
Flow cytometry analysis apoptosis of HepG2 cells in different groups. Q1‐LL, living cells; Q1‐UR, late apoptotic cells; Q1‐LR, early apoptotic cells.

The apoptosis of HepG2 cells was shown in Figure 2. Early apoptosis was 0.30%, and late apoptosis was 4.26% in HepG2 group. Early apoptosis was 1.67% and late apoptosis was 15.4% in HepG2 + autoblood group. Early apoptosis was 0.18% and late apoptosis was 7.22% in HepG2 + autoblood + inhibitor group. The apoptosis rate was the highest in the HepG2 + autoblood group, followed by the HepG2 + autoblood + inhibitor group, indicating that autologous blood transfusion could effectively promote the death of tumour cells.

### 3.3 Autophagosomes observation by transmission electron microscopy

**FIGURE 3:**
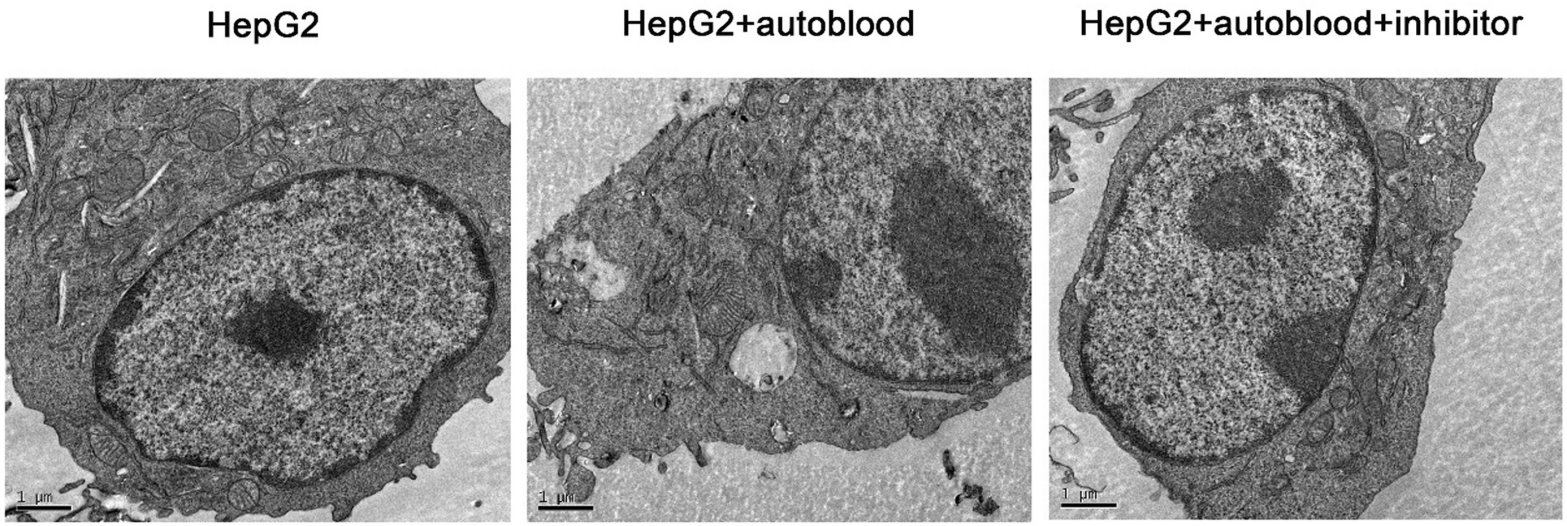
Autophagosomes were observed by transmission electron microscopy (×1 μm).

The autophagosomes with vesicular structure were observed by transmission electron microscopy, as shown in Figure 3. The number of autophagosomes in HepG2 + autoblood was the largest, and the second was HepG2 + autoblood + inhibitor group.

### 3.4 HIF‐1α expression of immunofluorescence assay

**FIGURE 4:**
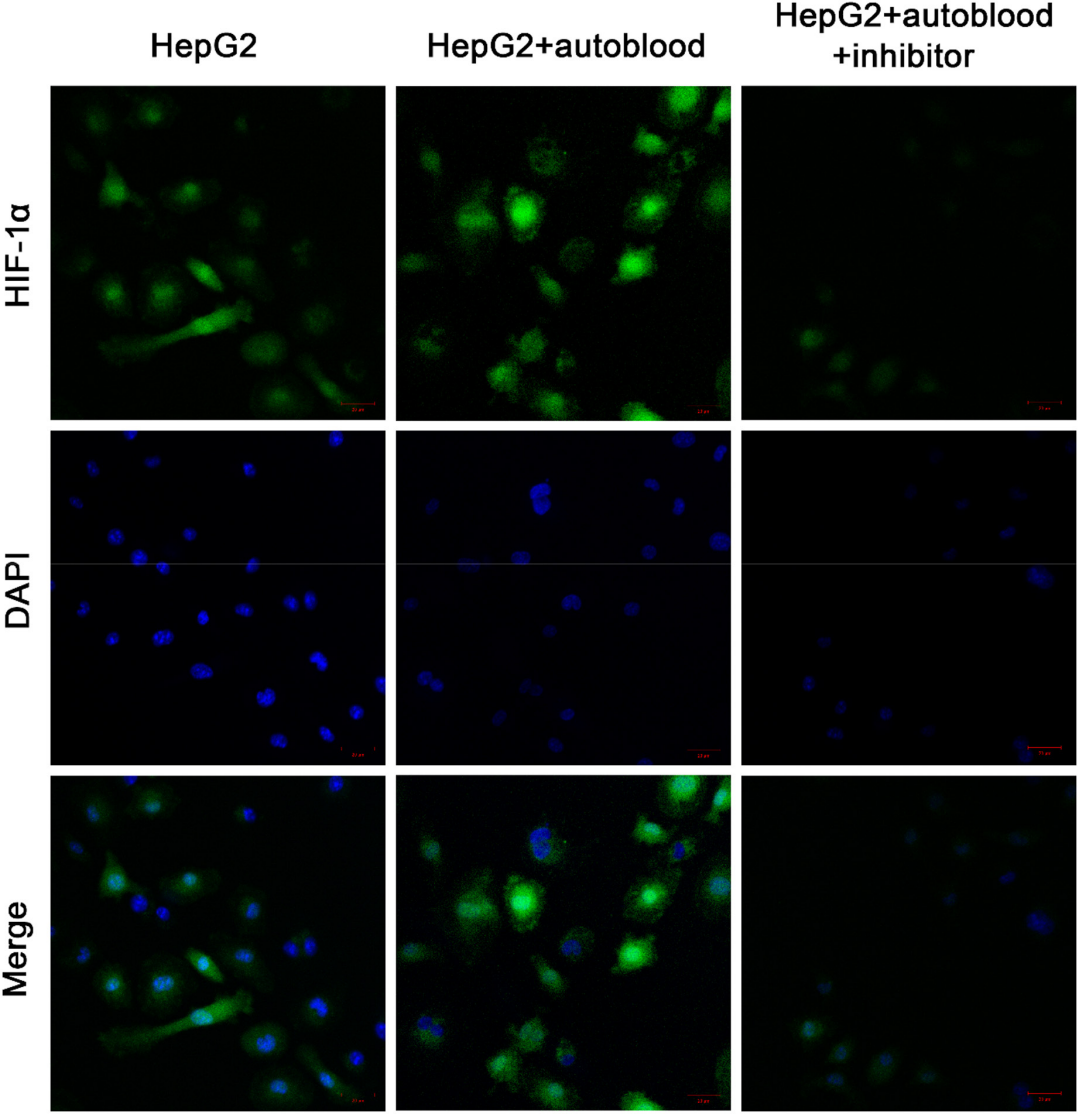
The relative expression of HIF‐1α protein was analysed by immunofluorescence assay (×200).

HepG2 cells of each group were collected, and the relative expression level of HIF‐1α protein in cells was analysed by immunofluorescence assay, as shown in Figure 4. Green fluorescence showed HIF‐1α protein expression, while blue fluorescence showed DAPI stained cell nucleus. High expression of HIF‐1α was clearly observed in HepG2 and HepG2 + autoblood group from confocal observation. However, there was no HIF‐1α protein expression in HepG2 + autoblood + inhibitor group. This is because Oltipraz can inhibit HIF‐1α protein expression in cells.

### 3.5 Migration and invasion of HepG2 cells

**FIGURE 5:**
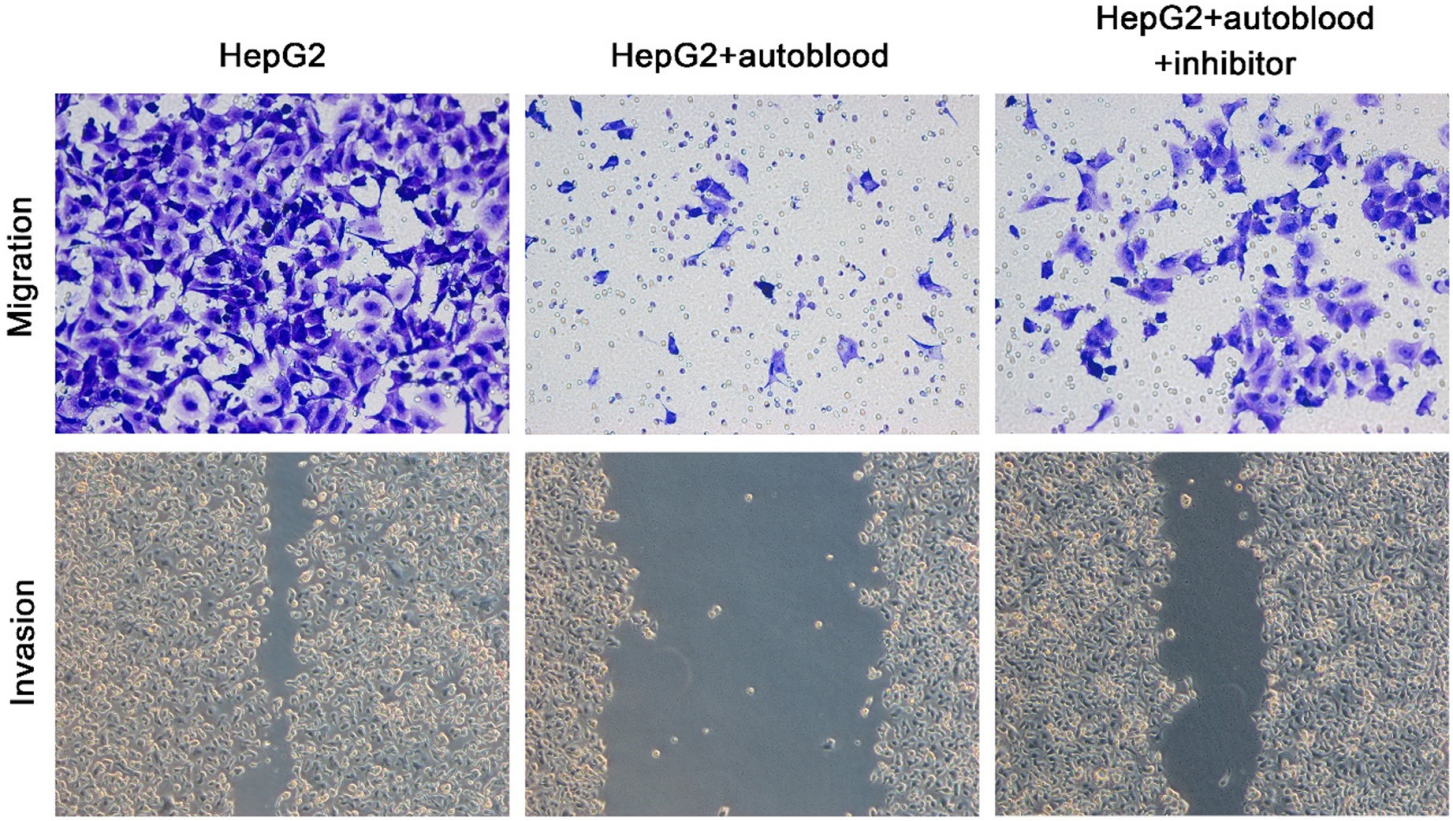
The migration and invasion of HepG2 cells in different groups (×400).

HepG2 cells in each group were collected, and the migration and invasion abilities of cells were analysed by transwell and scratch test, as shown in Figure 5. Compared with HepG2 group, the number of cells decreased significantly in HepG2 + autoblood group and HepG2 + autoblood + inhibitor group. Among of them, HepG2 + autoblood group was the least. It indicates that the invasion ability of cells in HepG2 + autoblood group was weakest. The migration rate in HepG2 group, HepG2 + autoblood group and HepG2 + autoblood + inhibitor group was 85.71 ± 7.38%, 14.36 ± 6.54% and 61.25 ± 5.39%, respectively. Compared with HepG2 group, the migration rate of HepG2 + autoblood group decreased much, and there was a significant difference, *p* < 0.001.

### 3.6 Western blot analysis of HIF‐1α, TP53, MDM2, ATG5 and ATG14 expression in HepG2 cells

**FIGURE 6:**
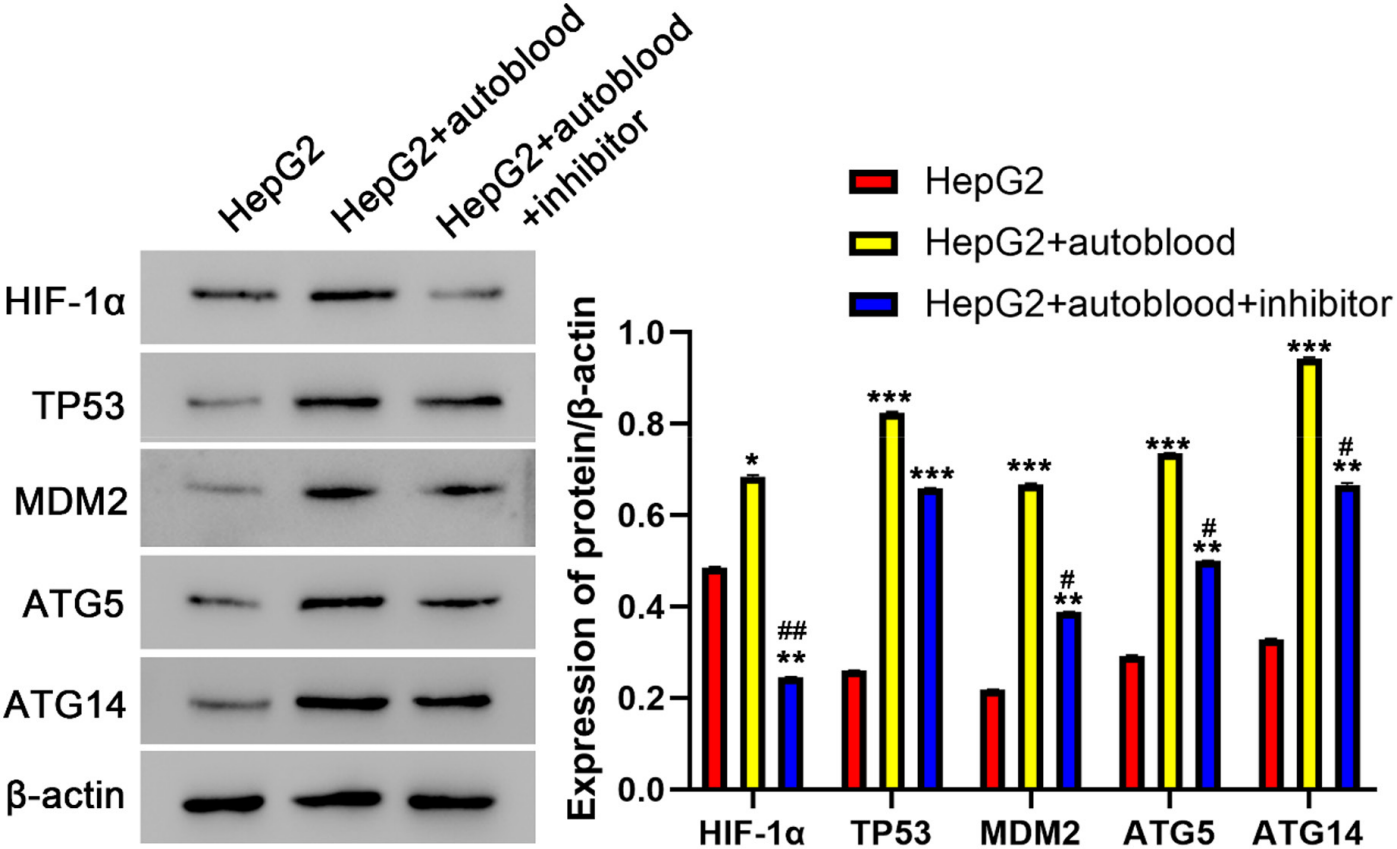
Protein levels of HIF‐1α, TP53, MDM2, ATG5 and ATG14 in different groups determined by Western blot (Mean ± SD, *n* = 3). *p < 0.05; **p < 0.01; ***p < 0.001 compared with HepG2 group; ^*##*^ *p* < 0.01; ^*#*^ *p* < 0.05 compared with HepG2 + autoblood group.

The expressions of HIF‐1α, TP53, MDM2, ATG5 and ATG14 proteins in HepG2 cells were showed in Figure 6. The content of HIF‐1α, TP53, MDM2, ATG5 and ATG14 was all highest in model + autoblood group. Secondly, HIF‐1α content of model group was higher, but content of TP53, MDM2, ATG5 and ATG14 in the model group is the second. Compared with HepG2 group, *p < 0.05; **p < 0.01; ***p < 0.001. Compared with model + autoblood group, ^*##*^ *p* < 0.01; ^*#*^ *p* < 0.05.

## 4 DISCUSSION

Blood transfusion is an important means to ensure the safety of operation and reduce ischemic injury of viscera. In patients with liver cancer, surgical treatment is the most important among various treatments. However, due to the complex anatomical location of the liver and its rich blood supply system result in difficult operation.^16^ Sometimes, the operation is extremely complex, and severe bleeding may occur during the operation. Therefore, only a large amount of blood transfusion is possible to ensure the smooth operation and postoperative recovery. At present, the most commonly used methods of blood transfusion include allogeneic blood transfusion and autologous blood transfusion. In addition, allogeneic blood transfusion may cause allergic and rejection reactions, as well as immunosuppression and tumour proliferation.^17^, ^18^, ^19^ Transfusions of concentrated red blood cells and frozen plasma are the most common in autotransfusion for intraoperative haemorrhage.

The role of autophagy in tumours is considered to be bidirectional: on the one hand, autophagy can degrade useless proteins and organelles, reduce oxidative stress of cells, inhibit chronic tissue damage and prevent the occurrence of tumours. On the other hand, autophagy can provide metabolic energy needed by tumour cells to promote their growth and survival by degrading and recycling intracellular substances under unfavourable circumstances.^20^ Moreover, studies have proved that autophagy has analgesic effects, such as autophagy promoting aging^21^ and autophagy mediating programmed cell death.^22^ At the same time, many studies in genetically engineered mice have confirmed that the deletion of the At96/Beclinl allele of autophagy regulatory gene induces liver cancer, and the deletion of Atg5 or Atg7 can also cause liver cancer in mice.^23^ In this study, it was found that the number of autophagy was the highest in the HepG2 + autoblood group, and the apoptosis rate of HepG2 cells was the highest (16.07%), which was significantly higher than that in the HepG2 group. The number of autophagy decreased in the HepG2 + autoblood HIF‐1α inhibitor group. It suggests that autophagy can inhibit the proliferation of liver cancer cells through HIF‐1α signalling pathway.

Hypoxia‐inducible factor‐1α (HIF‐1α) is a transcription factor widely present in mammals and humans under hypoxia conditions. HIF‐1α is also a key factor in response to hypoxia stress. It is regulated by hypoxia and regulates the activity of HIF‐1.^24^ Under hypoxia, HIF‐1α is transferred into the nucleus and binds to HIF‐1β to form active HIF‐1, which regulates transcription of multiple genes by binding to hypoxia response elements on target genes. HIF‐1α can form different signalling pathways with a variety of upstream and downstream proteins, mediate hypoxia signal, regulate a series of compensatory responses to hypoxia, and play an important role in physiological and pathological processes of the body. In this work, we found that the expression of HIF‐1α in normal rat liver tissue was significantly lower than that in model group, while that in model group was lower than that in autotransfusion group. HIF‐1α expression of immunofluorescence assay showed that high expression of HIF‐1α was clearly observed in HepG2 and HepG2 + autoblood group from confocal observation. However, there was no HIF‐1α protein expression in HepG2 + autoblood + inhibitor group. It suggests that autologous blood transfusion can increase the expression of HIF‐1α during hepatocellular carcinoma surgery. The relative expression of HIF‐1α was consistent with that of rat liver. In addition, the results of cell migration and invasion assay confirmed that the HepG2 + autoblood group were the weakest.

## 5 CONCLUSION

Rat and cell experiments have demonstrated that autologous blood transfusion can enhance the expression of related proteins in the HIF‐1α signalling pathway. Moreover, the number of autophagosomes in HepG2 cells was significantly the largest after autologous blood transfusion, resulting in apoptosis. It suggests that autologous blood transfusion promotes autophagy of HCC cells through HIF‐1α signalling pathway, thus further inhibiting HCC migration and erosion.

## AUTHOR CONTRIBUTIONS

**Yu Bai:** Conceptualization (equal); data curation (equal); investigation (equal); methodology (equal); supervision (equal); validation (equal); visualization (equal); writing – original draft (equal); writing – review and editing (equal). **Tong Liu:** Conceptualization (equal); data curation (equal); investigation (equal); methodology (equal); software (equal); supervision (equal); visualization (equal); writing – original draft (equal); writing – review and editing (equal). **Ying‐Hui Cui:** Data curation (equal); formal analysis (equal); methodology (equal); supervision (equal); validation (equal); visualization (equal); writing – original draft (equal); writing – review and editing (equal). **Zhen‐Zhou Li:** Formal analysis (equal); investigation (equal); methodology (equal); supervision (equal); visualization (equal); writing – review and editing (equal). **Xiao‐Fang Zhou:** Formal analysis (equal); investigation (equal); methodology (equal); software (equal); validation (equal); writing – review and editing (equal). **Yong Cheng:** Formal analysis (equal); investigation (equal); methodology (equal); software (equal); validation (equal); writing – review and editing (equal). **Jin‐Huo Wang:** Data curation (equal); formal analysis (equal); investigation (equal); validation (equal); visualization (equal); writing – review and editing (equal). **Jian‐Rong Guo:** Formal analysis (equal); investigation (equal); supervision (equal); validation (equal); writing – review and editing (equal).

## FUNDING INFORMATION

This work is supported by National Natural Science Foundation of China (no. 82170225) and Key Disciplines Group Construction Project of Pudong Health Bureau of Shanghai (Grant no. PWZxq2022‐5).

## CONFLICT OF INTEREST STATEMENT

All of the authors have no conflict of interest in this research.

## Data Availability

The data are available if necessary.
